# Elevated 1-hour Post Load Glucose as a Predictor for Telomere Attrition: A Study Based on a Chinese Community Population

**DOI:** 10.1210/clinem/dgae748

**Published:** 2024-10-23

**Authors:** Qi Gao, Jie Yu, Yiwen Liu, Baodi Xing, Fan Ping, Lingling Xu, Wei Li, Huabing Zhang, Yuxiu Li

**Affiliations:** Department of Endocrinology, Key Laboratory of Endocrinology of National Health Commission, Translation Medicine Center, Peking Union Medical College Hospital, Chinese Academy of Medical Sciences and Peking Union Medical College, Beijing, 100730, China; Department of Endocrinology, Key Laboratory of Endocrinology of National Health Commission, Translation Medicine Center, Peking Union Medical College Hospital, Chinese Academy of Medical Sciences and Peking Union Medical College, Beijing, 100730, China; Department of Endocrinology, Key Laboratory of Endocrinology of National Health Commission, Translation Medicine Center, Peking Union Medical College Hospital, Chinese Academy of Medical Sciences and Peking Union Medical College, Beijing, 100730, China; Department of Endocrinology, Key Laboratory of Endocrinology of National Health Commission, Translation Medicine Center, Peking Union Medical College Hospital, Chinese Academy of Medical Sciences and Peking Union Medical College, Beijing, 100730, China; Department of Endocrinology, Key Laboratory of Endocrinology of National Health Commission, Translation Medicine Center, Peking Union Medical College Hospital, Chinese Academy of Medical Sciences and Peking Union Medical College, Beijing, 100730, China; Department of Endocrinology, Key Laboratory of Endocrinology of National Health Commission, Translation Medicine Center, Peking Union Medical College Hospital, Chinese Academy of Medical Sciences and Peking Union Medical College, Beijing, 100730, China; Department of Endocrinology, Key Laboratory of Endocrinology of National Health Commission, Translation Medicine Center, Peking Union Medical College Hospital, Chinese Academy of Medical Sciences and Peking Union Medical College, Beijing, 100730, China; Department of Endocrinology, Key Laboratory of Endocrinology of National Health Commission, Translation Medicine Center, Peking Union Medical College Hospital, Chinese Academy of Medical Sciences and Peking Union Medical College, Beijing, 100730, China; Department of Endocrinology, Key Laboratory of Endocrinology of National Health Commission, Translation Medicine Center, Peking Union Medical College Hospital, Chinese Academy of Medical Sciences and Peking Union Medical College, Beijing, 100730, China

**Keywords:** glycemic parameters, 1-hour post-load plasma glucose, aging, leukocyte telomere length, telomere attrition

## Abstract

**Context:**

One-hour post-load glucose (1h-PG) detects dysglycemia-related disorders more effectively than traditional glycemic parameters. Hyperglycemia accelerates aging, but whether 1h-PG outperforms in predicting aging remains unclear.

**Objective:**

To compare the effectiveness of 1h-PG with other glycemic parameters in identifying and predicting telomere attrition.

**Methods:**

We conducted a cross-sectional and longitudinal study based on a Chinese community cohort. Multivariate linear regression and logistic regression were used to analyze the associations between glycemic parameters and telomere length. The area under the receiver operating characteristic (AUROC) curve were used to compare the differentiating and predictive ability. Populations were regrouped by glucose tolerance status and 1h-PG to compare telomere length. Analyses were separately conducted in nondiabetic and diabetic populations.

**Results:**

The cross-sectional study included 715 participants. Only 1h-PG was significantly negatively associated with relative telomere length in both nondiabetic [β = −.106, 95% confidence interval (CI) −0.068 to −0.007, *P* = .017] [odds ratio (OR) = 1.151, 95% CI 1.069 to 1.239, *P* = .005] and diabetic (β = −.222, 95% CI −0.032 to −0.007, *P* = .002) (OR = 1.144, 95% CI 1.041 to 1.258, *P* = .035) populations. The longitudinal study recruited 437 populations and 112 remained in 7-years follow-up. 1h-PG was associated with telomere shortening in the nondiabetic group (β = −.314, 95% CI −0.276 to −0.032, *P* = .016) (OR = 2.659, 95% CI 1.158 to 6.274, *P* = .021). AUROC analysis showed that 1h-PG outperformed other glycemic parameters in identifying and predicting telomere attrition. Reclassification revealed that normal glucose tolerance and prediabetic individuals with elevated 1h-PG had telomere lengths comparable to prediabetic and diabetic populations, respectively.

**Conclusion:**

1h-PG outperforms other glycemic parameters in predicting telomere attrition and can be a valuable marker for early aging detection.

Aging, marked by the progressive decline in function due to molecular and cellular deterioration (1), is now recognized as the predominant risk factor for major chronic diseases, including cancer, cardiovascular disorders, dementia, and, ultimately, increased mortality (2). The World Health Organization has launched the Decade of Healthy Aging 2020-2030 initiative, aiming to ameliorate societal aging processes (3). Telomeres, which cap the ends of eukaryotic chromosomes, play a crucial role in safeguarding DNA from damage (4). Recent findings underscore that telomere shortening is pivotal in regulating cellular aging, with leukocyte telomere length (LTL) emerging as a significant aging biomarker (5). Understanding factors that accelerate telomere attrition is essential for developing strategies to slow the aging process.

Recent research has elucidated a significant correlation between plasma glucose levels and LTL. For example, LTL in diabetic (DM) patients is shorter than their age-matched but nondiabetic (non-DM) counterparts (6). The LTL in islet β-cell is inverse to the level of glycated hemoglobin (HbA1c) in DM populations (7). Shortened LTL may present a marker of glucose deterioration (8). However, traditional glycemic parameters showed instability in predicting telomere shortening and may differ among populations with varying glucose tolerance statuses (9). It is necessary to identify more effective glycemic biomarkers for detecting aging.

Increasing evidence indicates that elevated 1-hour post-load plasma glucose (1h-PG) exhibits greater sensitivity than traditional glycemic parameters such as fasting plasma glucose (FPG), 2-hour post-load plasma glucose (2h-PG), and HbA1c in identifying dysglycemia-related conditions (10). A recent study from Daqing, China, utilizing 30 years of follow-up data, revealed that non-DM individuals with 1h-PG ≥8.6 mmol/L (1 mmol/L = 18 mg/dL) had a significantly increased risk of developing diabetes [hazard ratio (HR) = 4.45, 95% confidence interval (CI) 3.43 to 5.79, *P* < 0.001] (11). Recently, the International Diabetes Federation issued a statement advocating that 1h-PG ≥ 8.6 mmol/L for intermediate hyperglycemia diagnosis and ≥11.6 mmol/L for DM, confirming its value in early dysglycemia detection (12). Within populations exhibiting normal glucose tolerance (NGT), an elevated 1h-PG has been shown to be a strong predictor for the complications of diabetes and increased mortality risk (13-15). To our knowledge, there were no previous studies that have explored whether 1h-PG is superior to traditional glycemic parameters in identifying and predicting telomere attrition.

Utilizing data from a Chinese community cohort, we analyzed the relationship between various glycemic parameters (FPG, 30′-PG, 1h-PG, 2h-PG, and HbA1c) and LTL in a cross-sectional study, and compared their ability to predict telomere shortening over a 7-year follow-up.

## Material and Methods

### Participants

The data were derived from a cohort in the Changping district of Beijing, China. The purpose of establishing the cohort is to explore the factors influencing the occurrence and progression of diabetes and its related diseases. Therefore, it includes individuals with NGT, prediabetes, and diabetes. Participants were initially recruited between March 2014 and January 2015, with follow-ups conducted every 1 to 2 years. Additionally, new participants were dynamically enrolled and regularly monitored throughout this period. Comprehensive assessments were performed at baseline and during each follow-up. All individuals provided written informed consent voluntarily and underwent a standardized 75 g oral glucose tolerance test. Additionally, they completed questionnaires detailing their sex, age, medical history, smoking, alcohol consumption, and exercise status.

The diagnostic criteria for categorizing glucose tolerance levels adhered to the 1999 World Health Organization standards (16), wherein (1) NGT is defined as FPG < 6.1 mmol/L and 2h-PG < 7.8 mmol/L; (2) impaired fasting glucose (IFG) as FPG ≥ 6.1 mmol/L but < 7.0 mmol/L with 2h-PG < 7.8 mmol/L; (3) impaired glucose tolerance (IGT) as FPG <7.0

 mmol/L and 2h-PG ≥ 7.8 mmol/L but < 11.1 mmol/L; (4) DM as FPG ≥7.0 mmol/L or 2h-PG ≥11.1 mmol/L; (5) the non-DM population includes individuals with NGT, IFG, and IGT; (6) prediabetic (pre-DM) population encompasses both IFG and IGT.

**Figure 1.**
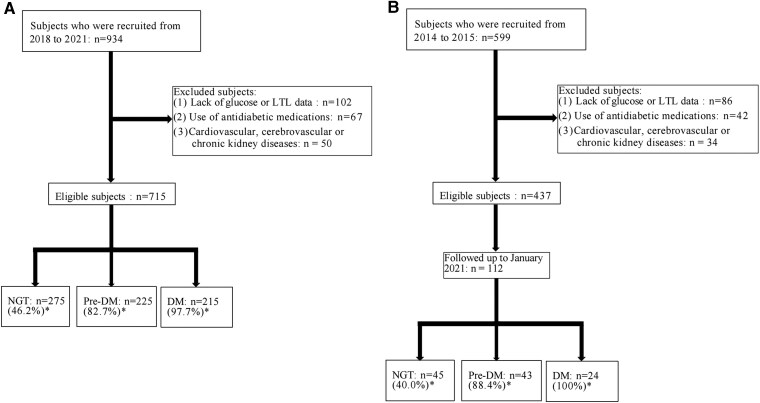
Flowchart of participants included in the study. (1A) Flowchart of cross-sectional study; (1B) flowchart of prospective study. *Proportion of individuals with elevated 1-hour plasma glucose (≥8.6 mmol/L). Abbreviations: DM, diabetic group; LTL, leukocyte telomere length; NGT, normal glucose tolerance; Pre-DM, prediabetic group.

In the cross-sectional study, we used data from 934 participants collected between 2018 and 2021. Exclusion criteria were consistent with baseline, and 715 participants were eligible for final analysis. In the total population, the numbers of individuals with NGT, prediabetes, and diabetes are 275, 225, and 215, respectively. Additionally, the number and proportion of individuals with elevated 1h-PG (≥8.6 mmol/L) in these 3 glucose tolerance categories are as follows: 127 (46.2%) for NGT, 186 (82.7%) for prediabetes, and 210 (97.7%) for diabetes (Fig. 1A).

In the prospective study, we recruited 599 adults at the baseline. Exclusion criteria included (1) recent (within the past 3 months) use of steroids, antidiabetic medications, or other agents potentially affecting plasma glucose levels; (2) cardiovascular and cerebrovascular diseases or chronic kidney diseases; (3) participants without the data of glucose or LTL. After applying these criteria, 437 participants were eligible for recruitment, and there were 112 participants remaining when followed up to 2021. In the total 112 individuals, the numbers of individuals with NGT, prediabetes, and diabetes were 45, 43, and 24, respectively. Moreover, the number and percentage of individuals with 1h-PG ≥8.6 mmol/L in the 3 glucose tolerance groups were as follows: 18 (40.0%) for NGT, 38 (88.4%) for prediabetes, and 24 (100%) for diabetes (Fig. 1B).

The study's protocols received approval from the Ethics Committee of Peking Union Medical College Hospital.

### LTL Assays

LTL assays were conducted using blood samples, with the methodology for LTL assessment detailed in a prior publication (17). In summary, LTL was quantified by calculating the ratio of telomere repeat copy number to single gene copy number (T/S ratio) using a monochrome multiplex quantitative PCR method (18). The efficiencies of the standard curves for both primer sets exceeded 90%, with regression coefficients of at least 0.99 across all PCR runs. The coefficient of variation (CV) within and between plates, calculated as the ratio of the SD to the mean across replicates, were 18% and 7%, respectively. For the study samples, the within-plate CV ranged from 8.2% to 14.3%.

In the cross-sectional study, the relative telomere length (RTL) of leukocytes served as a proxy for telomere length. RTL below the median (RTL ≤ P50) indicates a group with shorter telomeres. For the longitudinal study, RTL was measured at both baseline and follow-up. To mitigate potential batch effects on RTL measurements over time, RTL values were log-transformed and standardized to Z-scores by subtracting the mean and dividing by the SD, thus normalizing individual LTL measurements to the cohort mean (19). Z-scores of logs-transformed LTL at baseline (Z-scores _BL_) and follow-up (Z-scores _FU_) were separately calculated. The change of Z-scores (ΔZ-scores) were calculated as Z-scores _FU_ minus Z-scores _BL_. Accelerated telomere attrition was defined as ΔZ-scores below the median(ΔZ-scores ≤ P50).

Given the various terms used to refer to telomere length in this study, we provide the following definitions and explanations for clarity: (1) LTL: telomere length. This broadly refers to the length of telomeres; (2) RTL: relative telomere length. LTL was quantified by calculating the ratio of telomere repeat copy number to single gene copy number (T/S ratio) using a monochrome multiplex quantitative PCR method. RTL represents the actual calculated telomere length used in the analysis of the cross-sectional study; (3) Z-scores of RTL: RTL was standardized using Z-scores to mitigate potential batch effects on RTL measurements over time in the longitudinal study; (4) Z-scores _BL_: Z-scores standardization of RTL at baseline; (5) Z-scores _FU_: Z-scores standardization of RTL at follow-up; (6) ΔZ-scores: the difference between Z-scores of RTL at follow-up and baseline (Z-scores _FU_ minus Z-scores _BL_). This is used to assess the extent of telomere shortening in the longitudinal study.

### LTL Comparison by Glucose Tolerance Status and 1h-PG Levels

Recently, the International Diabetes Federation guidelines recommended 1h-PG thresholds of 8.6 mmol/L for prediabetes and 11.6 mmol/L for diabetes diagnosis (12). These cut-off values are based on large longitudinal studies showing 1h-PG ≥8.6 mmol/L predicts future diabetes risk in individuals with NGT (20-23). Additionally, a meta-analysis of 15 studies with 35 551 participants found 1h-PG ≥11.6 mmol/L has higher sensitivity and specificity for diabetes compared to the 2h-PG ≥11.1 mmol/L (24). Thus, using elevated 1h-PG for diagnosis may enable earlier treatment when glycemic control is easier and diabetes reversal more likely. However, it remains unclear whether these new diagnostic cut-off values can differentiate telomere length among different glucose tolerance status groups. Therefore, we examined whether the NGT but with elevated 1h-PG (≥8.6 mmol/L) population has telomere lengths comparable to those in the pre-DM population, and whether pre-DM with elevated 1h-PG levels (≥11.6 mmol/L) match telomere lengths in the DM group.

### Anthropometric Examination

Participants underwent height and weight assessments in light clothing and without shoes. Body mass index (BMI) was calculated as the individual's weight in kilograms divided by the square of their height in meters. Measurements of waist circumference (WC) and hip circumference (HC), along with systolic blood pressure (SBP) and diastolic blood pressure (DBP), were conducted twice by trained clinicians. WC was accurately measured to the nearest 0.1 cm at the midpoint between the iliac crest and the costal margin on both sides. Similarly, HC was determined to the nearest 0.1 cm around the hip rotor.

### Dietary Assessment

Daily energy intake was evaluated through 24-hour food recalls, with dietary data gathered by trained interviewers. Nutrient intakes were computed utilizing nutrition calculation software, developed by registered dietitians and based on the Microsoft Office Access 2007 database. The nutrient consumption calculations were guided by the China Food Composition Table 2004 database.

### Biochemical Measurements

Following an overnight fast of at least 10 hours, participants received a 2-hour 75 g oral glucose tolerance test in the morning. Plasma glucose levels were measured at 0, 30, 60, and 120 minutes using the glucose oxidase method. HbA1c was analyzed via high-performance liquid chromatography with intra-assay CV < 3% and interassay CV <10%. Total cholesterol, triglycerides (TG), high-density lipoprotein cholesterol (HDL-C), low-density lipoprotein cholesterol (LDL-C), uric acid (UA), creatinine (Cr), aspartate aminotransferase (AST), and alanine aminotransferase were quantified using an automated analyzer.

### Statistical Analysis

The Shapiro–Wilk and Kolmogorov–Smirnov tests were utilized to assess the normality of the data distribution. Variables conforming to a normal distribution were presented as mean ± SD, whereas those not normally distributed were shown as median with interquartile range (25th-75th percentile). Categorical variables were shown as counts and percentages. Group differences were evaluated using one-way ANOVA, Student's *t*-test, Mann–Whitney U test, or chi-squared test, as appropriate.

In the cross-sectional analysis, RTL was divided into tertiles for comparing participant characteristics. The relationships between glycemic parameters and RTL were assessed through multiple linear regression models, adjusted for age, sex, BMI, WC, HC, SBP, plasma lipids (TG, LDL-C, HDL-C), AST, UA, Cr, and lifestyle-related factors (smoking, alcoholic drinking, daily energy intake, exercise). The ability of glycemic parameters to distinguish shorter RTL was evaluated using multivariable binary logistic regression with the same adjusted confounders as in the linear regression, and the effectiveness was measured using the area under the receiver operating characteristic (AUROC) curve.

For the longitudinal analysis, Z-scores _BL_ and Z-scores _FU_ were calculated, with the ΔZ-scores (Z-scores _FU_ minus Z-scores _BL_) representing the change in LTL over 7-year period. ΔZ-scores below the median (ΔZ-scores ≤ P50) indicates a group with significantly accelerate telomere attrition. Multiple linear regression analyzed the association between baseline glycemic parameters and ΔZ-scores, adjusting for age, sex, BMI, WC, HC, SBP, TG, LDL-C, HDL-C, AST, UA, Cr, daily energy intake, and Z-scores _BL_. Participants were stratified into 2 groups based on their ΔZ-scores being equal to or less than or greater than the median, and baseline characteristics were compared between the 2 groups. The predictive capacity of glycemic parameters for accelerated telomere shortening was assessed using multivariable binary logistic regression and the AUROC curve, with Youden's index determining optimal glycemic parameter cutoffs.

All statistical tests were two-tailed, with a *P* value of less than .05 deemed statistically significant. Statistical analyses were performed using SPSS software, version 26.0 (IBM Corp.), and graphical representations were generated with GraphPad Prism version 10.0 (available at https://www.graphpad.com).

## Results

### Participant Characteristics

In the cross-sectional study, we compared the demographic and clinical features of the study population across tertiles of RTL (Table 1). Among the 715 participants, the age was 57.0 ± 10.0 years, with males comprising 35.1% of the population, and the RTL was observed at 0.8 (0.6, 1.1). There were 215 (30.1%) DM individuals in the total population. A significant trend emerged with ascending RTL tertiles: individuals tended to be younger and exhibit reduced WC, HC, and TG levels alongside higher HDL-C levels. Notably, all glycemic parameters assessed in this study demonstrated a declining trend from the lowest to the highest RTL tertile. Additionally, the lowest RTL tertile group has a higher proportion of DM individuals compared to the highest RTL tertile group.

**Table 1.** Characteristics of the participants in the cross-sectional study

Variable		RTL tertile			*P*
	All (n = 715)	T1 (n = 239)	T2 (n = 238)	T3 (n = 238)	
Glucose tolerance status (% diabetes)	215 (30.1)	97 (40.6)*^c^*	67 (28.2)	51 (21.4)*^a^*	
RTL	0.8 (0.6, 1.1)	0.5 (0.4, 0.6)*^b,c^*	0.8 (0.7, 0.9)*^a,c^*	1.3 (1.1, 1.5)*^a,b^*	.001
Age, years	57.0 ± 10.0	59.0 ± 9.0*^b,c^*	57.0 ± 9.0*^a^*	55.0 ± 11.0*^a^*	.000
Sex (% male)	251 (35.1)	92 (38.5)	90 (37.8)	69 (29.0)	.053
Smoking, n (%)	342 (47.8)	121 (50.6)	120 (50.4)	101(42.4)	.619
Alcohol drinking, n (%)	356 (49.8)	119 (49.79)	122 (51.3)	115 (48.31)	.856
Regular exercise, n (%)	350 (49.0)	107 (44.8)	124 (52.1)	119 (50.0)	.256
Energy intake, kcal/d	1624.1 ± 468.1	1583.9 ± 473.0	1616.7 ± 473.7	1672.1 ± 455.2	.115
BMI, kg/m^2^	26.1 ± 4.5	26.4 ± 3.4	25.9 ± 3.9	25.9 ± 5.7	.388
WC, cm	88.7 ± 11.1	90.5 ± 11.9*^c^*	88.5 ± 9.9	87.3 ± 11.3*^a^*	.004
HC, cm	99.7 ± 11.8	100.5 ± 11.0*^c^*	99.1 ± 8.9	97.7 ± 14.1*^a^*	.030
WHR	0.90 (0.85, 0.94)	0.91 (0.86, 0.94)	0.89 (0.85, 0.94)	0.89 (0.84, 0.93)	.490
SBP, mmHg	132.0 ± 42.0	134.0 ± 17.0	135.0 ± 66.0	128.0 ± 24.0	.114
DBP, mmHg	78.0 (71.0, 81.0)	79.0 (71.0, 88.0)	75.0 (73.0, 86.0)	77.0 (70.0, 86.0)	.260
ALT, U/L	16.0 (13.0, 24.0)	15.0 (12.0, 22.0)	17.0 (13.0, 25.0)	16.5 (13.0, 25.0)	.483
AST, U/L	21.0 (17.0, 25.0)	20.0 (17.0, 24.0)	21.0 (18.0, 25.0)	21.0 (17.0, 25.0)	.763
Cr, μmmol/L	66.0 (57.0, 76.0)	66.0 (56.0, 76.0)	66.0 (59.0, 76.0)	65.0 (57.0, 75.0)	.494
UA, μmmol/L	298.4 ± 77.7	298.0 ± 81.9	296.1 ± 77.0	301.1 ± 74.1	.777
TG, mmol/L	3.3 (1.7, 4.8)	3.9 (2.2, 4.9)*^b,c^*	2.9 (1.2, 4.8)*^a^*	2.7 (1.4, 4.6)*^a^*	.001
TC, mmol/L	2.8 (1.3, 4.7)	3.7 (1.5, 4.9)	2.9 (1.3, 4.7)	1.9 (1.1, 4.7)	.141
HDL-C, mmol/L	1.3 ± 0.3	1.2 ± 0.3*^c^*	1.3 ± 0.3	1.4 ± 0.3*^a^*	.039
LDL-C, mmol/L	2.9 (2.3, 3.4)	2.9 (2.3, 3.4)	2.9 (2.4, 3.4)	2.8 (2.3, 3.4)	.304
FPG, mmol/L	6.5 ± 1.9	7.0 ± 2.3*^b,c^*	6.3 ± 1.8*^a^*	6.0 ± 1.5*^a^*	.000
30′-PG, mmol/L	10.3 ± 3.3	11.0 ± 3.6*^c^*	10.4 ± 8.1*^c^*	9.5 ± 7.7*^a,b^*	.000
1h-PG, mmol/L	12.0 ± 4.8	13.8 ± 5.1*^b,c^*	11.3 ± 4.7*^a,c^*	10.8 ± 3.9*^a,b^*	.000
2h-PG, mmol/L	10.1 ± 5.5	11.6 ± 6.1*^c^*	9.9 ± 5.5*^c^*	9.0 ± 4.6*^a,b^*	.000
HbA1c, %	6.1 ± 1.2	6.3 ± 1.4*^c^*	6.0 ± 1.2	5.9 ± 1.0*^a^*	.038

Data are presented as mean ± SD, median (25th-75th percentile), and number (%).

*P* < .05 indicates statistical difference.

Abbreviations: 1h-PG, 1-hour post-load plasma glucose; 2h-PG, 2-hour post-load plasma glucose; 30′-PG, 30-min post-load plasma glucose; ALT, alanine aminotransferase; AST, aspartate aminotransferase; BMI, body mass index; Cr, creatinine; DBP, diastolic blood pressure; FPG, fasting plasma glucose; HbA1c, glycated hemoglobin; HC, hip circumstance; HDL-C, high-density lipoprotein cholesterol; LDL-C, low-density lipoprotein cholesterol; RTL, relative telomere length; SBP, systolic blood pressure; T, tertiles; TC, total cholesterol; TG, triglyceride; UA, uric acid; WC, waist circumference; WHR, waist-to-hip ratio.

^*a*^A significant difference compared with T1.

^*b*^A significant difference compared with T2.

^*c*^A significant difference compared with T3.

In the prospective study, 437 patients were eligible with 112 completing the follow-up from 2014 to 2021. The cohort predominantly consisted of males (68.8%); the age was 53 ± 8 years at baseline. The ΔZ-scores were −0.3 (−1.0, 0.9), while the Z-scores _BL_ were 0.00 (−0.68, 0.71). Compared with ΔZ-scores > P50 group, participants with a ΔZ-scores ≤ P50 exhibited higher FPG (6.7 ± 1.8 vs 6.2 ± 1.2), 30′-PG (12.1 ± 3.3 vs 10.6 ± 2.7), 1h-PG (13.1 ± 4.9 vs 9.9 ± 3.7), and 2h-PG (9.9 ± 5.0 vs 7.7 ± 3.6), though HbA1c levels did not differ significantly between groups (6.0 ± 1.0 vs 5.8 ± 0.8). There were also higher baseline Z-scores (0.62 [0.25, 1.21] vs −0.68 [−0.98, −0.25]) in ΔZ-scores ≤ P50 group. Significant differences were not observed between the 2 groups for age, anthropometric measures, plasma lipid levels, and daily energy intake (Table 2).

**Table 2.** Baseline characteristics in the prospective study

Variable	All (n = 112)	ΔZ-scores ≤ P50 (n = 56)	ΔZ-scores > P50 (n = 56)	*P*
ΔZ-scores	−0.3 (−1.0, 0.9)	−1.0 (−1.5, −0.5)	1.5 (1.2, 1.8)	.000
Z-scores _BL_	0.00 (−0.68, 0.71)	0.62 (0.25, 1.21)	−0.68 (−0.98, −0.25)	.000
Age, years	53.0 ± 8.0	52.0 ± 7.0	53.0 ± 9.0	.690
Sex (% male)	77 (68.8)	40 (71.4)	37 (66.1)	.541
Energy intake, kcal/d	1730.5 ± 584.2	1770.4 ± 568.3	1689.3 ± 607.0	.592
BMI, kg/m^2^	26.3 ± 4.1	26.3 ± 4.5	26.3 ± 3.7	.999
WC, cm	88.0 ± 10.4	88.8 ± 10.6	87.2 ± 10.3	.422
HC, cm	93.1 ± 10.8	93.7 ± 11.1	92.6 ± 10.5	.583
WHR	0.95 ± 0.02	0.95 ± 0.02	0.94 ± 0.02	.064
SBP, mmHg	129.5 ± 18.6	131.0 ± 20.0	128.0 ± 18.0	.256
DBP, mmHg	77.1 ± 9.4	78.0 ± 10.0	76.0 ± 9.0	.165
ALT, U/L	25.2 (19.8, 36.3)	24.9 (19.8, 36.6)	25.4 (19.7, 33.3)	.351
AST, U/L	24.2 (19.6, 28.0)	25.2 (19.6, 29.3)	23.4 (19.6, 26.8)	.407
Cr, μmmol/L	68.1 (57.7, 81.6)	69.1 (58.7, 81.3)	66.9 (56.5, 82.9)	.834
UA, μmmol/L	283.3 ± 72.0	286.1 ± 63.5	280.5 ± 80.0	.684
TG, mmol/L	1.4 (1.0, 1.9)	1.6 (1.1, 2.3)	1.4 (1.0, 1.9)	.145
TC, mmol/L	5.5 ± 0.9	5.5 ± 0.8	5.5 ± 1.1	.882
HDL-C, mmol/L	1.3 ± 0.3	1.3 ± 0.2	1.3 ± 0.3	.553
LDL-C, mmol/L	2.8 ± 0.7	2.9 ± 0.6	2.9 ± 0.7	.959
FPG, mmol/L	6.5 ± 1.6	6.7 ± 1.8	6.2 ± 1.2	.011
30′-PG, mmol/L	11.4 ± 3.1	12.1 ± 3.3	10.6 ± 2.7	.007
1h-PG, mmol/L	11.5 ± 4.6	13.1 ± 4.9	9.9 ± 3.7	.000
2h-PG, mmol/L	8.8 ± 4.5	9.9 ± 5.0	7.7 ± 3.6	.005
HbA1c, %	5.9 ± 0.9	6.0 ± 1.0	5.8 ± 0.8	.121

Data are presented as mean ± SD, median (25th-75th percentile), and number (%).

RTL at baseline and follow-up were transformed to Z-scores; ΔZ-scores were calculated by Z-scores _FU_ minus Z-scores _BL_; ΔZ-scores below the median (≤P50) represent accelerated telomere attrition.

*P* < .05 indicates statistical difference.

Abbreviations: 1h-PG, 1-hour post-load plasma glucose; 2h-PG, 2-hour post-load plasma glucose; 30**′**-PG, 30-min post-load plasma glucose; ALT, alanine aminotransferase; AST, aspartate aminotransferase; BMI, body mass index; Cr, creatinine; DBP, diastolic blood pressure; FPG, fasting plasma glucose; HbA1c, glycated hemoglobin; HC, hip circumstance; HDL-C, high-density lipoprotein cholesterol; LDL-C, low-density lipoprotein cholesterol; RTL, relative telomere length; SBP, systolic blood pressure; TC, total cholesterol; TG, triglyceride; UA, uric acid; WC, waist circumference; WHR, waist-to-hip ratio; Z-scores _BL_, Z-scores at baseline; Z-scores _FU_, Z-scores at follow-up.

### Association of RTL With Glycemic Parameters in Different Populations

In the cross-sectional study, a multiple linear regression analysis was conducted to determine the independent impact of glycemic parameters on RTL (Table 3). In the total population, only p load glucose including 30′-PG (β = −.086, 95% CI −0.040 to −0.002, *P* = .027), 1h-PG (β = −.143, 95% CI −0.037 to −0.012, *P* = .000), and 2h-PG (β = −.093, 95% CI −0.025 to −0.003, *P* = .015) retained a significant negative correlation with RTL after adjusting for confounders. When classifying the population into non-DM and DM groups, only 1h-PG showed a significant inverse correlation with RTL, even after full adjustment for confounders (β = −.106, 95% CI −0.068 to −0.007, *P* = .017; β = −.222, 95% CI −0.032 to −0.007, *P* = .002 in the non-DM and DM group, respectively).

**Table 3.** Multivariate linear regression between RTL and glycemic parameters in the cross-sectional study

	FPG		30′-PG		1h-PG		2h-PG		HbA1c	
	β (95% CI)	*P*	β (95% CI)	*P*	β (95% CI)	*P*	β (95% CI)	*P*	β (95% CI)	*P*
Total population										
Model 1	−.133 (−0.085; −0.025)	.000	−.125 (−0.049; −0.013)	.001	−.191 (−0.044; −0.020)	.000	−.140 (−0.031; −0.010)	.000	−.085 (−0.104; −0.007)	.024
Model 2	−.117 (−0.079; −0.018)	.002	−.109 (−0.045; −0.009)	.004	−.174 (−0.042; −0.017)	.000	−.122 (−0.028; −0.007)	.001	−.075 (−0.097; −0.001)	.045
Model 3	−.104 (−0.075; −0.012)	.006	−.101 (−0.043; −0.006)	.008	−.160 (−0.040; −0.015)	.000	−.109 (−0.027; −0.005)	.004	−0.067 (−0.093; 0.005)	.078
Model 4	−.075 (−0.063; 0.000)	.051	−.086 (−0.040; −0.002)	.027	−.143 (−0.037; −0.012)	.000	−.093 (−0.025; −0.003)	.015	−.042 (−0.078; 0.022)	.268
Non-DM group										
Model 1	−.051 (−0.217; 0.058)	.258	−.037 (−0.066; 0.027)	.406	−.139 (−0.080; −0.018)	.002	−.066 (−0.079; 0.011)	.138	.039 (−0.068; 0.137)	.390
Model 2	−.026 (−0.182; 0.099)	.561	−.028 (−0.061; 0.031)	.527	−.128 (−0.076; −0.014)	.004	−.058 (−0.074; 0.015)	.272	.041 (−0.063; 0.176)	.354
Model 3	−.021 (−0.178; 0.111)	.648	−.036 (−0.066; 0.028)	.427	−.118 (−0.073; −0.010)	.009	−.050 (−0.071; 0.020)	.272	.032 (−0.082; 0.170)	.490
Model 4	−.023 (−0.180; 0.106)	.612	−.007 (−0.050; 0.043)	.880	−.106 (−0.068; −0.007)	.017	−.037 (−0.060; 0.031)	.411	.058 (−0.064; 0.026)	.240
DM group										
Model 1	−.140 (−0.142; −0.001)	.040	−.117 (−0.025; 0.002)	.087	−.219 (−0.031; −0.008)	.001	−.083 (−0.015; 0.003)	.225	−.120 (−0.062; 0.003)	.078
Model 2	−.142 (−0.042; −0.001)	.038	−.110 (−0.025; 0.003)	.107	−.210 (−0.030; −0.007)	.002	−.071 (−0.014; 0.004)	.303	−.126 (−0.063; 0.002)	.065
Model 3	−.109 (−0.037; 0.003)	.101	−.063 (−0.020; 0.007)	.350	−.182 (−0.028; −0.004)	.007	−.056 (−0.013; 0.005)	.411	−.092 (−0.054; 0.010)	.170
Model 4	−.082 (−0.034; 0.008)	.234	−.102 (−0.025; 0.004)	.150	−.222 (−0.032; −0.007)	.002	−.112 (−0.017; 0.002)	.116	−.122 (−0.063; 0.002)	.069

Model 1: unadjusted; model 2: adjusted for age and sex; model 3: model 2 + BMI, WC, HC, SBP, smoking, alcohol drinking, energy intake, exercise status; model 4: model 3 + TG, LDL-C, HDL-C, AST, UA, Cr.

*P* < .05 indicates statistical difference.

Abbreviations: 1h-PG, 1-hour post-load plasma glucose; 2h-PG, 2-hour post-load plasma glucose; 30**′**-PG, 30-min post-load plasma glucose; ALT, alanine aminotransferase; BMI, body mass index; Cr, creatinine; CI, confidence interval; DM, diabetic; FPG, fasting plasma glucose; HbA1c, glycated hemoglobin; HC, hip circumstance; HDL-C, high-density lipoprotein cholesterol; LDL-C, low-density lipoprotein cholesterol; non-DM, nondiabetic; RTL, relative telomere length; SBP, systolic blood pressure; TG, triglyceride; UA, uric acid; WC, waist circumference;

Subsequently, we utilized multivariable binary logistic regression to determine whether glycemic parameters are associated with an increased risk of shorter RTL (Table 4). In the general population, all glycemic parameters except HbA1c were associated with shorter RTL. In the non-DM group, after adjusting for confounders, only FPG (OR = 1.523, CI 1.093 to 2.123, *P* = .013), 1h-PG (OR = 1.151, CI 1.069 to 1.239, *P* = .005), and 2h-PG (OR = 1.126, CI 1.015 to 1.250, *P* = .025) were found to increase the risk of shorter RTL. In the DM group, only 1h-PG significantly increased the risk (OR = 1.144, CI 1.041 to 1.258, *P* = .035).

**Table 4.** Logistic regression between glycemic parameters and RTL ≤ P50 in the cross-sectional study

	FPG		30′-PG		1h-PG		2h-PG		HbA1c	
	OR (95% CI)	*P*	OR (95% CI)	*P*	OR (95% CI)		OR (95% CI)	*P*	OR (95% CI)	*P*
Total population										
Model 1	1.202 (1.102, 1.311)	.000	1.103 (1.051, 1.156)	.000	1.098 (1.062, 1.135)	.000	1.056 (1.027, 1.086)	.000	1.153(1.018, 1.305)	.025
Model 2	1.180 (1.083, 1.287)	.001	1.093 (1.042, 1.147)	.000	1.091 (1.055, 1.128)	.000	1.050 (1.021, 1.080)	.001	1.135(1.003, 1.286)	.045
Model 3	1.160 (1.062, 1.267)	.006	1.079 (1.028, 1.134)	.002	1.084 (1.048, 1.122)	.003	1.046 (1.016, 1.076)	.002	1.023(0.988, 1.278)	.076
Model 4	1.169 (1.067, 1.280)	.011	1.089 (1.135, 1.146)	.010	1.093 (1.055, 1.133)	.008	1.053 (1.022, 1.085)	.016	1.152(0.936, 1.319)	.091
Non-DM group										
Model 1	1.796 (1.317, 2.448)	.000	1.123 (1.015, 1.242)	.025	1.162 (1.083, 1.247)	.000	1.138 (1.031, 1.256)	.010	1.430(0.630, 1.094)	.187
Model 2	1.687 (1.106, 1.932)	.001	1.114 (1.006, 1.234)	.038	1.155 (1.075, 1.240)	.000	1.129 (1.022, 1.247)	.017	1.422(0.623, 1.085)	.167
Model 3	1.548 (1.121, 2.139)	.008	1.102 (0.993, 1.224)	.068	1.146 (1.066, 1.233)	.002	1.129 (1.019, 1.250)	.020	1.267(0.641, 1.173)	.356
Model 4	1.523 (1.093, 2.123)	.013	1.101 (0.989, 1.226)	.079	1.151 (1.069, 1.239)	.005	1.126 (1.015, 1.250)	.025	1.153(0.628, 1.160)	.311
DM group										
Model 1	1.118 (0.985, 1.269)	.085	1.071 (0.990, 1.160)	.089	1.111 (1.033, 1.196)	.005	1.035 (0.983, 1.090)	.190	1.170 (0.960, 1.424)	.119
Model 2	1.122 (0.988, 1.275)	.075	1.096 (0.987, 1.158)	.100	1.110 (1.032, 1.195)	.005	1.033 (0.981, 1.089)	.220	1.179 (0.968, 1.436)	.102
Model 3	1.104 (0.960, 1.270)	.166	1.041 (0.955, 1.136)	.362	1.104 (1.017, 1.199)	.018	1.026 (0.969, 1.087)	.379	1.144 (0.919, 1.422)	.228
Model 4	1.156 (0.958, 1.306)	.156	1.071 (0.973, 1.180)	.163	1.144 (1.041, 1.258)	.035	1.052 (0.984, 1.126)	.138	1.290 (0.922, 1.661)	.148

RTL below the median (≤P50) represents the shorter RTL group.

Model 1: unadjusted; model 2: adjusted for age and sex; model 3: model 2 + BMI, WC, HC, SBP, smoking, alcohol drinking, energy intake, exercise status; model 4: model 3 + TG, LDL-C, HDL-C, AST, UA, Cr.

*P* < .05 indicates statistical difference.

Abbreviations: 1h-PG, 1-hour post-load plasma glucose; 2h-PG, 2-hour post-load plasma glucose; 30**′**-PG, 30-min post-load plasma glucose; ALT, alanine aminotransferase; BMI, body mass index; CI, confidence interval; Cr, creatinine; DM, diabetic; FPG, fasting plasma glucose; HbA1c, glycated hemoglobin; HC, hip circumstance; HDL-C, high-density lipoprotein cholesterol; LDL-C, low-density lipoprotein cholesterol; non-DM, nondiabetic; OR, odds ratio; RTL, relative telomere length; SBP, systolic blood pressure; TG, triglyceride; UA, uric acid; WC, waist circumference.

In the prospective study, we analyzed the correlation between glycemic parameters and ΔZ-scores using multiple linear regression (Table 5). In the total population, after adjusting for confounders, only post-load glucose parameters including 30′-PG (β = −.230, CI −0.360 to −0.027, *P* = .032), 1h-PG (β = −.255, CI −0.121 to −0.034, *P* = .005), and 2h-PG (β = −.156, CI −0.089 to −0.003, *P* = .035) showed significant associations. In the non-DM group, 1h-PG remained related to ΔZ-scores (β = −.314, CI −0.276 to −0.032, *P* = .016) after controlling for age, sex, BMI, WC, HC, SBP, daily energy intake, plasma lipids, UA, Cr, and Z-scores _BL_. No correlations reached statistical difference in the diabetes group, although 1h-PG showed the lowest *P*-value (β = −.146, CI −0.077 to 0.022, *P* = .220).

**Table 5.** Multivariate linear regression between ΔZ-scores and glycemic parameters in the prospective study

	FPG		30′-PG		1h-PG		2h-PG		HbA1c	
	β (95% CI)	*P*	β (95% CI)	*P*	β (95% CI)	*P*	β (95% CI)	*P*	β (95% CI)	*P*
Total population										
Model 1	−.187 (−0.384; −0.001)	.049	−.251 (−0.225; −0.036)	.008	−.401 (−0.201; −0.080)	.000	−.280 (−0.166; −0.036)	.003	−.281 (−0.731; −0.060)	.021
Model 2	−.187 (−0.387; −0.000)	.049	−.250 (−0.226; −0.034)	.008	−.400 (−0.201; −0.079)	.000	−.277 (−0.166; −0.034)	.003	−.216 (−0.729; −0.052)	.024
Model 3	−.127 (−0.360; 0.146)	.400	−.318 (−0.258; −0.014)	.030	−.466 (−0.222; −0.062)	.001	−.305 (−0.174; −0.006)	.030	−.148 (−0.641; 0.199)	.296
Model 4	−.121 (−0.232; 0.027)	.118	−.230 (−0.360; −0.027)	.032	−.255 (−0.121; −0.034)	.005	−.156 (−0.089; −0.003)	.035	−.179 (−0.477; 0.056)	.115
Non-DM group										
Model 1	−.074 (−0.920; 0.446)	.492	−.175 (−0.310; 0.029)	.103	−.426 (−0.374; −0.020)	.000	−.321 (−0.525; −0.118)	.002	−.151 (−1.226; 0.209)	.362
Model 2	−.077 (−0.938; 0.446)	.482	−.185 (−0.323; 0.026)	.094	−.427 (−0.378; −0.139)	.000	−.319 (−0.526; −0.313)	.003	−.148 (−1.231; 0.231)	.178
Model 3	−.160 (−1.679; 0.809)	.479	−.594 (−0.591; 0.093)	.074	−.704 (−0.511; −0.182)	.001	−.518 (−0.814; −0.145)	.007	−.155 (−1.321; 0.611)	.456
Model 4	−.194 (−1.125; 0.069)	.080	−.375 (−0.330; 0.020)	.112	−.314 (−0.276; −0.032)	.016	−.099 (−0.320; 0.136)	.407	−.176 (−0.865; 0.055)	.081
DM group										
Model 1	.051 (−0.189; 0.237)	.814	−.019 (−0.143; 0.131)	.929	−.175 (−0.145; 0.062)	.415	.019 (−0.082; 0.019)	.928	−.053 (−0.393; 0.309)	.807
Model 2	.067 (−0.197; 0.262)	.770	.079 (−0.128; 0.077)	.740	−.130 (−0.140; 0.078)	.560	.094 (−0.073; 0.109)	.682	−.050 (−0.406; 0.327)	.823
Model 3	−.011 (−0.115; 0.107)	.931	−.081 (−0.090; 0.052)	.537	−.146 (−0.077; 0.022)	.220	−.124 (−0.071; 0.030)	.362	−.023 (−0.224; 0.192)	.861

RTL at baseline and follow-up were transformed to Z-scores; ΔZ-scores were calculated by Z-scores _FU_ minus Z-scores _BL_.

Model 1: unadjusted; model 2: adjusted for age and sex; model 3: model 2 + BMI, WC, HC, SBP, energy intake (for DM group, model 3 was adjusted for age, sex, and Z-scores _BL_); model 4: model 3 + TG, LDL-C, HDL-C, AST, UA, Cr, Z-scores _BL_.

*P* < .05 indicates statistical difference.

Abbreviations: 1h-PG, 1-hour post-load plasma glucose; 2h-PG, 2-hour post-load plasma glucose; 30**′**-PG, 30-min post-load plasma glucose; ALT, alanine aminotransferase; BMI, body mass index; CI, confidence interval; Cr, creatinine; DM, diabetic; FPG, fasting plasma glucose; HbA1c, glycated hemoglobin; HC, hip circumstance; HDL-C, high-density lipoprotein cholesterol; LDL-C, low-density lipoprotein cholesterol; non-DM, nondiabetic; RTL, relative telomere length; SBP, systolic blood pressure; TG, triglyceride; UA, uric acid; WC, waist circumference; Z-scores _BL_, Z-scores at baseline; Z-scores _FU_, Z-scores at follow-up.

Next, binary logistic regression was utilized to evaluate the predictive power of baseline glycemic parameters for accelerated telomere attrition (Table 6). In the total population, 30′-PG (OR = 1.761, CI 1.076 to 2.881, *P* = .024) and 1h-PG (OR = 1.776, 95% CI 1.051 to 2.571, *P* = .042) significantly predicted telomere shortening after adjusting for confounders. The impact of 2h-PG lessened (OR = 1.328, 95% CI 0.962 to 1.834, *P* = .084) after adjusting for plasma lipids and Z-scores _BL_. In the non-DM population, only 1h-PG at baseline significantly predicted telomere shortening (OR = 2.659, 95% CI 1.158 to 6.247, *P* = .021), even fully adjusting for confounders. No significant predictive effects were observed in the DM group.

**Table 6.** Logistic regression between glycemic parameters and ΔZ-scores ≤ P50 in the prospective study

	FPG		30′-PG		1h-PG		2h-PG		HbA1c	
	OR (95%CI)	*P*	OR (95%CI)	*P*	OR (95%CI)		OR (95%CI)	*P*	OR (95%CI)	*P*
Total population										
Model 1	1.248 (0.961, 1.622)	.097	1.182 (1.034, 1.967)	.014	1.205 (1.086, 1.337)	.000	1.135 (1.026, 1.256)	.014	1.418(0.903, 2.228)	.130
Model 2	1.246 (0.958, 1.621)	.101	1.188 (1.037, 1.360)	.013	1.207 (1.087, 1.341)	.000	1.134 (1.025, 1.254)	.015	1.411(0.896, 2.222)	.137
Model 3	1.312 (0.990, 1.339)	.059	1.195 (1.041, 1.371)	.011	1.212 (1.088, 1.349)	.000	1.150 (1.035, 1.277)	.009	1.391(0.881, 2.197)	.157
Model 4	1.616 (0.883, 2.958)	.120	1.761 (1.076, 2.881)	.024	1.776 (1.051, 2.571)	.042	1.328 (0.962, 1.834)	.084	1.854 (0.703, 4.890)	.212
Non-DM group										
Model 1	1.160 (0.526, 2.560)	.712	1.178 (0.957, 1.449)	.123	1.304 (1.091, 1.560)	.004	1.207 (0.934, 1.559)	.150	0.824 (0.352, 1.926)	.655
Model 2	1.183 (0.533, 2.624)	.680	1.198 (0.966, 1.485)	.100	1.307 (1.091, 1.566)	.004	1.201 (0.929, 1.553)	.161	0.802 (0.340, 1.891)	.614
Model 3	1.318 (0.562, 2.088)	.525	1.196 (0.934, 1.463)	.173	1.292 (1.066, 1.566)	.009	1.217 (0.920, 1.608)	.168	0.614 (0.224, 1.678)	.341
Model 4	1.599 (0.451, 2.967)	.285	1.187 (0.948, 1.272)	.124	2.659 (1.158, 6.274)	.021	1.287 (0.707, 2.343)	.410	0.191 (0.007, 5.122)	.824
DM group										
Model 1	1.031 (0.679, 1.564)	.887	1.098 (0.834, 1.444)	.506	1.168 (0.932, 1.463)	.178	1.082 (0.908, 1.288)	.378	1.197 (0.597, 2.397)	.613
Model 2	0.958 (0.596, 1.539)	.859	0.993 (0.723, 1.364)	.964	1.146 (0.896, 1.464)	.277	1.041 (0.861, 1.260)	.677	1.153 (0.529, 2.512)	.720
Model 3	0.794 (0.224, 2.813)	.720	0.998 (0.546, 1.825)	.994	1.079 (0.687, 1.693)	.741	0.980 (0.669, 1.436)	.919	1.164 (0.540, 2.173)	.780

RTL at baseline and follow-up were transformed to Z-scores; ΔZ-scores were calculated by Z-scores _FU_ minus Z-scores _BL_; ΔZ-scores below the median (≤P50) represent accelerated telomere attrition.

Model 1: unadjusted; model 2: adjusted for age and sex; model 3: model 2 + BMI, WC, HC, SBP, energy intake (for DM group, model 3 was adjusted for age, sex, and Z-scores _BL_); model 4: model 3 + TG, LDL-C, HDL-C, AST, UA, Cr, Z-scores _BL_.

*P* < .05 indicates statistical difference.

Abbreviations: 1h-PG, 1-hour post-load plasma glucose; 2h-PG, 2-hour post-load plasma glucose; 30**′**-PG, 30-min post-load plasma glucose; ALT, alanine aminotransferase; BMI, body mass index; CI, confidence interval; Cr, creatinine; DM, diabetic; FPG, fasting plasma glucose; HbA1c, glycated hemoglobin; HC, hip circumstance; HDL-C, high-density lipoprotein cholesterol; LDL-C, low-density lipoprotein cholesterol; non-DM, nondiabetic; OR, odds ratio; RTL, relative telomere length; SBP, systolic blood pressure; TG, triglyceride; UA, uric acid; WC, waist circumference; Z-scores _BL_, Z-scores at baseline; Z-scores _FU_, Z-scores at follow-up.

### The Identifying and Predictive Abilities of Glycemic Parameters for Telomere Attrition

**Figure 2.**
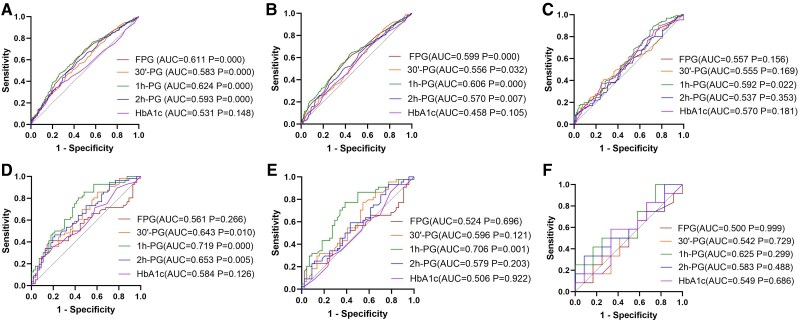
AUROCs of the glycemic parameters for telomere attrition. (2A) Total population in cross-sectional study; (2B) non-DM population in cross-sectional study; (2C) DM population in cross-sectional study; (2D) total population in prospective study; (2E) non-DM population in prospective study; (2F) DM population in prospective study. *P* < .05 indicates statistical difference. Abbreviations: 1h-PG, 1-hour post-load plasma glucose; 2h-PG, 2-hour post-load plasma glucose; 30′-PG, 30-minute post-load plasma glucose; AUROC, area under the receiver operating characteristic; DM, diabetic; FPG, fasting plasma glucose; HbA1c, glycated hemoglobin; non-DM, nondiabetic.

In the cross-sectional study, AUROC results showed the ability of glycemic parameters to identify shorter RTL. For the total and non-DM populations, all glycemic parameters except HbA1c could identify shorter RTL, with 1h-PG exhibited the strongest identifying capability (AUC = 0.624, *P* < .001; AUC = 0.606, *P* < .001 for the total and non-DM populations, respectively)(Fig. 2A and 2B), with optimal cutoff at 12.5 mmol/L (47.4% sensitivity, 73.1% specificity) for the total population and 9.05 mmol/L (63.2% sensitivity, 65.8% specificity) for the non-DM population. In the DM group, only 1h-PG showed the potential (AUC = 0.592, *P* = .022), with the best cutoff at 13.8 mmol/L (sensitivity 89.8%, specificity 37.3%) (Fig. 2C).

In the longitudinal study, AUROC analysis revealed the predictive capabilities of glycemic parameters for accelerated telomere attrition. In the total population, post-load glucose levels demonstrated significant predictive ability, and 1h-PG had the highest predictive value with an AUROC of 0.719 (*P* < .001) with the optimal cutoff at 9.2 mmol/L (sensitivity: 77.3%, specificity: 62.8%) (Fig. 2D). In the non-DM group, only 1h-PG showed the predictive ability with an AUROC of 0.706 (*P* = .001), and the best cutoff was 9.0 mmol/L (sensitivity: 77.3%, specificity: 62.8%) (Fig. 2E). No significant predictive differences were observed in the DM group, with the highest AUROC for 1h-PG at 0.625 (*P* = .299) (Fig. 2F).

### Comparison of RTL Across Groups Sorted by Glucose Tolerance Status and 1h-PG

**Figure 3.**
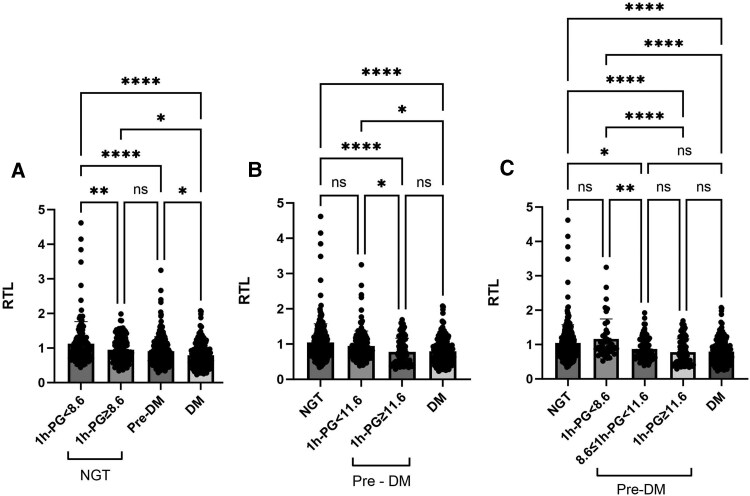
Comparison of LTL across groups sorted by glucose tolerance status and 1h-PG. (3A) Comparison of RTL across NGT with 1h-PG < 8.6 mmol/L, NGT with 1h-PG ≥ 8.6 mmol/L, pre-DM, and DM groups; (3B) comparison of RTL across NGT, pre-DM with 1h-PG < 11.6 mmol/L, pre-DM with 1h-PG ≥ 11.6 mmol/L, and DM groups; (3C) comparison of RTL across NGT, pre-DM with 1h-PG < 8.6 mmol/L, pre-DM with 8.6 ≤ 1h-PG < 11.6 mmol/L, pre-DM with 1h-PG ≥ 11.6 mmol/L, and DM groups. *P < .05; **P < .01; ****P < .0001; *P* < .05 indicates statistical difference. The vertical axis units in panels 3A, 3B, and 3C are the same, representing the level of RTL. Abbreviations: 1h-PG, 1-hour post-load plasma glucose; DM, diabetic group; NGT, normal glucose tolerance; ns, no significance; pre-DM, prediabetic group; RTL, relative telomere length.

First, we categorized NGT individuals into 2 groups based on 1h-PG levels (<8.6 mmol/L and ≥8.6 mmol/L) and assessed RTL differences across NGT with low 1h-PG (<8.6 mmol/L), NGT with high 1h-PG (≥8.6 mmol/L), pre-DM, and DM groups. The results showed that NGT with high 1h-PG individuals exhibited significantly shorter RTL compared to those with low 1h-PG. Furthermore, the RTL in the NGT with the high 1h-PG group paralleled that of the pre-DM group but was longer than that observed in the DM group (Fig. 3A).

Next, we categorized the pre-DM population based on 1h-PG into 2 groups: 1h-PG < 11.6 mmol/L and 1h-PG ≥ 11.6 mmol/L. We then compared the RTL among NGT, pre-DM with low 1h-PG (<11.6 mmol/L), pre-DM with high 1h-PG (≥11.6 mmol/L), and DM groups. The results showed that pre-DM with low 1h-PG had RTL comparable to the NGT group, while those with high 1h-PG showed an RTL match with the DM group (Fig. 3B).

Finally, we further subdivided the pre-DM participants based on their 1h-PG levels into 3 groups: 1h-PG < 8.6 mmol/L, 8.6 ≤ 1h-PG < 11.6 mmol/L, and 1h-PG ≥ 11.6 mmol/L. We compared the RTL of these 3 groups with those of the NGT and DM groups. The results showed that the RTL of the pre-DM group with 1h-PG between 8.6 and 11.6 mmol/L was significantly shorter than that of the pre-DM group with 1h-PG below 8.6 mmol/L. Moreover, the RTL of the pre-DM group with 1h-PG < 8.6 mmol/L was similar to that of the NGT group, while the RTL levels of the pre-DM groups with 8.6 ≤ 1h-PG < 11.6 mmol/L and 1h-PG ≥ 11.6 mmol/L, as well as the DM group, were comparable (Fig. 3C).

**Figure 4.**
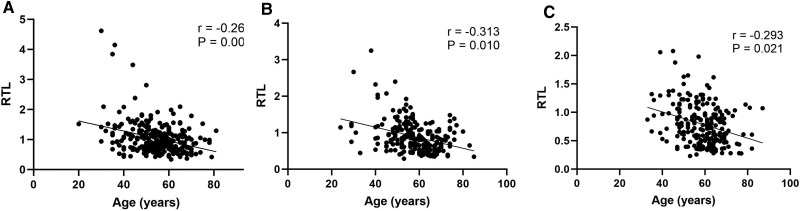
The association between age and RTL across different glucose tolerance groups. (A) Normal glucose tolerance group; (B) prediabetic group; (C) diabetic group. Spearman correlation analysis was used to examine the relationship between age and RTL. *P* < .05 indicates statistical difference. The vertical axis units in panels A, B, and C are the same, representing the level of RTL. Abbreviation: RTL, relative telomere length.

Additionally, to mitigate the impact of age differences on RTL levels across glucose tolerance groups, we compared their age distributions. The results showed no significant age differences, with mean ages of 55.0 ± 10.4 years for the NGT group, 57.0 ± 10.5 years for the pre-DM group, and 58.0 ± 8.3 years for the DM group. Spearman correlation analysis revealed a significant negative correlation between age and RTL in all 3 groups (Fig. 4).

## Discussion

By conducting a cross-sectional and prospective study based on a Chinese community cohort, we found that 1h-PG was more effective at identifying and predicting telomere shortening compared to other glycemic parameters, including 2h-PG. Additionally, NGT and pre-DM but with elevated 1h-PG populations showed telomere lengths comparable to pre-DM and DM populations, respectively.

Previous studies have illuminated a relationship between traditional glucose parameters and telomere length, yet the results were varied among different studies. Krasnienkov et al (9) identified that the correlations between FPG and 2h-PG with telomere length vary by glucose tolerance status and may be influenced by age. Moreover, the relationship between HbA1c and telomere length is not yet conclusively defined (7). These results implied that traditional glucose indicators may not be a stable marker for telomere attrition with their reliability potentially affected by metabolic and age-related factors. However, no previous studies have reported the relationship between 1h-PG and telomere length.

While research about the relationship between 1h-PG and telomere length is lacking, existing evidence shows that 1h-PG detects metabolic disorders more sensitively than traditional glycemic parameters. Individuals with NGT but elevated 1h-PG (≥8.6 mmol/L) showed high levels of oxidative stress, inflammation, and endothelial dysfunction, potentially escalating the risk of cardiovascular complications (25, 26). NGT individuals with 1h-PG ≥8.6 mmol/L are at increased risk for renal impairment and microalbuminuria (27). Furthermore, patients in the early stages of nonalcoholic fatty liver disease exhibit raised 1h-PG levels but normal FPG or 2h-PG (28). Moreover, a cohort study followed up for 30 years showed that non-DM individuals with a 1h-PG > 11.1 mmol/L exhibited a 1.49-fold increased risk for death compared with 1h-PG ≤11.1 mmol/L (29). To some extent, these studies provide support for our findings that 1h-PG may also have an advantage in screening for telomere shortening.

Mechanistically, existing research emphasizes that 1h-PG may better reflect pancreatic β-cells function. Studies have found that in individuals with NGT, an elevation in 1h-PG is associated with reductions in Matsuda's insulin sensitivity index and the oral disposition index, which respectively measure insulin sensitivity and β-cell function (30). Additionally, the Insulinogenic Index, a marker for assessing early-phase insulin secretion from β-cells, and the Homeostatic Model Assessment of Beta-cell Function both exhibit a stronger negative correlation with 1h-PG compared to other glycemic markers (31). This suggests that 1h-PG has a higher potential for detecting early changes in glucose metabolism.

Recently, several large-scale cohort studies from China have shown a close relationship between LTL and metabolic diseases, particularly diabetes (32). For example, Cheng et al (8), who conducted an 8.8-year follow-up study of 5506 Chinese patients with type 2 diabetes, revealed that shorter LTL predicts diabetes progression and glycemic deterioration. The authors also found that shorter LTL increases the risk of renal function decline, end-stage renal disease, and cardiovascular events in type 2 diabetes populations (33, 34). Our findings show a significant negative correlation between LTL and 1h-PG. Compared to other glucose parameters, elevated 1h-PG can more effectively identify the risk of telomere shortening earlier, even in non-DM individuals. Therefore, the reported advantage of 1h-PG in early detection of metabolic disorders may be related to its ability to detect early telomere shortening and aging risk.

### Strengths and Limitations

To the best of our knowledge, this is the first study demonstrating that 1h-PG better predicts telomere attrition than traditional glycemic parameters. We conducted both cross-sectional and longitudinal analyses and separately examined non-DM and DM populations to provide a more comprehensive interpretation.

The study has limitations. First, the longitudinal study had a low follow-up rate, primarily due to our reliance on voluntary participation for recruitment and recall, with some individuals possibly abstaining due to personal reasons. Second, in the longitudinal study, the results for the DM group were not statistically significant, possibly due to a small sample size. Further research with larger samples is needed to confirm these findings. Third, although we accounted for multiple significant confounding factors, the possibility of other influencing factors not being excluded cannot be dismissed. Fourth, our research was conducted in a Chinese population and may not be applicable to a broader demographic, warranting verification through studies in different ethnicities or regions.

## Conclusions

In summary, our study first found a significant negative correlation between 1h-PG and LTL and demonstrated a unique advantage over other glycemic parameters in predicting telomere attrition. This study broadens the predictive value of 1h-PG in aging, further providing substantial evidence about the superiority of 1h-PG in forecasting metabolic disorders compared to traditional glucose parameters.

## Data Availability

Restrictions apply to the availability of some, or all data generated or analyzed during this study to preserve patient confidentiality or because they were used under license. The corresponding author will on request detail the restrictions and any conditions under which access to some data may be provided.
